# Food Components and Dietary Habits: Keys for a Healthy Gut Microbiota Composition

**DOI:** 10.3390/nu11102393

**Published:** 2019-10-07

**Authors:** Emanuele Rinninella, Marco Cintoni, Pauline Raoul, Loris Riccardo Lopetuso, Franco Scaldaferri, Gabriele Pulcini, Giacinto Abele Donato Miggiano, Antonio Gasbarrini, Maria Cristina Mele

**Affiliations:** 1 UOC di Nutrizione Clinica, Dipartimento di Scienze Gastroenterologiche, Endocrino-Metaboliche e Nefro-Urologiche, Fondazione Policlinico Universitario A. Gemelli IRCCS, Largo A. Gemelli 8, 00168 Rome, Italy; giacintoabeledonato.miggiano@policlinicogemelli.it (G.A.D.M.); mariacristina.mele@unicatt.it (M.C.M.); 2 Istituto di Patologia Speciale Medica, Università Cattolica del Sacro Cuore, Largo F. Vito 1, 00168 Rome, Italy; pauline.raoul1@gmail.com (P.R.); antonio.gasbarrini@unicatt.it (A.G.); 3 Scuola di Specializzazione in Scienza dell’Alimentazione, Università di Roma Tor Vergata, Via Montpellier 1, 00133 Rome, Italy; marco.cintoni@gmail.com (M.C.); gabrielepulcini.med@gmail.com (G.P.); 4 UOC di Medicina Interna e Gastroenterologia, Dipartimento di Scienze Gastroenterologiche, Endocrino-Metaboliche e Nefro-Urologiche, Fondazione Policlinico Universitario A. Gemelli IRCCS, Largo A. Gemelli 8, 00168 Rome, Italy

**Keywords:** non-communicable diseases, leaky gut, gut microbiota modulation, diet, macronutrients, micronutrients, salt, food additives, low-calorie sweeteners, dietary emulsifiers, dietary habits, personalized medicine

## Abstract

The gut microbiota is a changing ecosystem, containing trillions of bacteria, continuously shaped by many factors, such as dietary habits, seasonality, lifestyle, stress, antibiotics use, or diseases. A healthy host–microorganisms balance must be respected in order to optimally maintain the intestinal barrier and immune system functions and, consequently, prevent disease development. In the past several decades, the adoption of modern dietary habits has become a growing health concern, as it is strongly associated with obesity and related metabolic diseases, promoting inflammation and both structural and behavioral changes in gut microbiota. In this context, novel dietary strategies are emerging to prevent diseases and maintain health. However, the consequences of these different diets on gut microbiota modulation are still largely unknown, and could potentially lead to alterations of gut microbiota, intestinal barrier, and the immune system. The present review aimed to focus on the impact of single food components (macronutrients and micronutrients), salt, food additives, and different dietary habits (i.e., vegan and vegetarian, gluten-free, ketogenic, high sugar, low FODMAP, Western-type, and Mediterranean diets) on gut microbiota composition in order to define the optimal diet for a healthy modulation of gut microbiota.

## 1. Introduction

The human gastrointestinal (GI) tract harbors more than 100,000 billion microorganisms, representing 10–100 times the number of human cells [1]. Bacteria are classified according to phyla, classes, orders, families, genera, and species. Only a few phyla are represented in the gut, accounting for more than 160 species [2]. The dominant gut microbial phyla are Firmicutes, Bacteroidetes, Actinobacteria, Proteobacteria, Fusobacteria, and Verrucomicrobia, with the two phyla Firmicutes and Bacteroidetes [3] representing 90% of gut microbiota. The Firmicutes phylum is composed of more than 200 different genera, such as *Lactobacillus, Bacillus, Clostridium, Enterococcus*, and *Ruminococcus*. *Clostridium* genera represent 95% of the Firmicutes phyla. Bacteroidetes consists of predominant genera such as *Bacteroides* and *Prevotella*. The Actinobacteria phylum is proportionally less abundant and mainly represented by the *Bifidobacterium* genus. There is no singular optimal gut microbiota composition since it is different for each individual [4]. Indeed, the human gut microbiota is characterized by an inter-individual variability due to infant transitions, antibiotics use, as well as lifestyle, dietary, and cultural habits. A rich and diverse microbial community leads to a well-balanced and healthy gut microbiota composition.

In recent years, many studies have shown the close association between gut microbiota dysbiosis and numerous non-communicable diseases, such as cardiovascular diseases [5], obesity [6], diabetes [7], cancer [8], gastrointestinal diseases such as irritable bowel syndrome (IBS) [9], and neurological disorders [10,11,12]. Furthermore, the reciprocal interactions between gut microbiota and the brain, well-known as the “gut–brain axis”, have been demonstrated through bidirectional neuroendocrine signaling and immune activations [13], playing a key role in several neurological disorders such as Parkinson’s disease [14] and autism spectrum disorders [15]. Although it is not entirely known if changes in gut microbiota composition are a cause or consequence of a given disease, the association between the richness and diversity of gut microbiota and health has been demonstrated [4]. 

Diet is one of the key modulators of gut microbiota composition which directly influences host homeostasis and biological processes but also via metabolites derived from the microbial fermentation of nutrients—particularly short-chain fatty acids (SCFAs) [16]. This crucial mutualism between the human host and its bacterial symbionts can be altered through novel dietary habits, potentially impacting intestinal barrier functions and the immune system. The present review aimed to explain how food components and dietary choices can modulate gut microbiota composition and, consequently, intestinal barrier functions. First, we describe the interplay between gut microbiota, intestinal barrier, and the immune system. Secondly, after defining the interactions between food components and gut microbiota, we focus on the relationship between dietary habits and gut microbiota to define the optimal dietary habits to shape a healthy microbiota composition and to maintain host gut barrier and immune functions. 

## 2. Gut Microbiota, Intestinal Barrier, and Immune System

A dynamic network orchestrates the interplay among the several components of the gastrointestinal tract, which is organized as a semipermeable multi-layer ecosystem [17,18,19,20]. A complex and mutualistic symbiosis regulates the relationship between the host and the gut microbiota [21,22,23]. This interplay is constantly challenged with numerous factors, and can lead to the collapse of the microbial community structure [17]. The intestinal barrier represents a functional unit responsible for two main tasks that are crucial for the survival of the individual: allowing nutrient absorption and defending the body from the penetration of unwanted, often dangerous, macromolecules. The gut mucosa is in fact a multi-layered system consisting of an external “anatomical” barrier and an inner “functional” immunological barrier [19]. Commensal gut microbiota, the mucous layer, and the intestinal epithelial monolayer constitute the anatomical barrier [21,22]. The deeper, inner layer consists of a complex network of immune cells organized in a specialized and compartmentalized system known as gut-associated lymphoid tissue (GALT). GALT represents both isolated and aggregated lymphoid follicles, and is one of the largest lymphoid organs, containing up to 70% of the body’s total number of immunocytes. Moreover, it is involved in the response to pathogenic microorganisms and provides immune tolerance to commensal bacteria. The ability of GALT to interact with luminal antigens rests on specific mucosal immune cells (i.e., dendritic cells and M-cells), primarily localized to Peyer’s patches within the ileum that are intimately positioned at the mucosa–environment interface and internalize microorganisms and macromolecules [17]. These specialized immune cells have the ability to present antigens to naïve T-lymphocytes, which subsequently produce cytokines and activate mucosal immune responses [23], when needed. Many factors such as alterations in the gut microflora, modifications of the mucus layer, and epithelial damage induced by the diet can alter this balance, leading to increased intestinal permeability and the translocation of luminal contents to the underlying mucosa [24]. The integrity of these structures is necessary for the maintenance of normal intestinal barrier function. The dysregulation of any of the aforementioned components has been implicated not only in the pathogenesis of IBD, but many other GI disorders, including infectious enterocolitis, irritable bowel syndrome, small intestinal bowel overgrowth, and allergic food intolerance [25,26,27]. In particular, several lines of evidence have shown that the microbial flora is critical for the development of a normal gut immune system, but can also play a central role in the development of IBD [28,29,30,31]. In support of this concept, the majority of genetically susceptible murine models of colitis do not develop significant inflammation when raised in a germ-free environment [32,33,34], while in others, diseases can be attenuated or completely abolished with antibiotic treatment [35,36]. In this context, innate immune responses that recognize conserved microbial products such as lipopolysaccharide (LPS) and peptidoglycan [37] are likely to be important in microbe–host interactions and intestinal homeostasis [38]. Critical to the host’s sensing of microbes are members of the toll-like receptor (TLR) family that, alone or in combination, recognize a wide array of microbe-associated molecular patterns (MAMPs) on either pathogenic or commensal microorganisms [39,40]. Furthermore, emerging lines of evidence indicate that intestinal homeostasis and inflammation are driven by cellular elements and soluble mediators that mediate both processes, with several cytokines exhibiting opposing roles depending upon the specific setting. In this context, all dietary components we introduce (or not introduce) may play a crucial role in shaping gut microbiota composition.

## 3. Interplays Between Food Components and Gut Microbiota

### 3.1. Carbohydrates and Gut Microbiota

#### 3.1.1. Carbohydrates

Carbohydrates can be categorized into digestible and indigestible substrates. Digestible carbohydrates such as glucose, fructose, and galactose are enzymatically degraded in the small intestine and rapidly released as glucose in the bloodstream. Conversely, indigestible carbohydrates, also called “dietary fiber” are resistant to digestion in the small intestine, and reach the large intestine. Dietary fibers include non-starch polysaccharides, lignin, resistant starches, and non-digestible oligosaccharides [41]. Non-starch polysaccharides include cellulose and hemicellulose (glucans, gums, and pectin). Resistant starch is commonly found within whole or partly milled grains or seeds. Finally, non-digestible oligosaccharides consist of raffinose, stachyose, oligofructose, and inulin. Dietary fibers may be categorized according to their fermentability (fermentable or non-fermentable) in the colon or to their solubility (soluble or insoluble) in water. Fermentable dietary fibers such as inulin, pectin, beta-glucan, fructo-oligosaccharides (FOSs), and galactooligosaccharides (GOSs) are considered water-soluble in nature, while non-fermentable dietary fibers such as cellulose, hemicellulose, lignin, and resistant starch are considered insoluble [42]. Fermentable fibers are easily fermented by bacteria in the colon, while non-fermentable fibers are not [42]. 

#### 3.1.2. SCFAs

Fermentable dietary fibers undergo saccharolytic fermentation, essentially under the action of gut bacteria, which in turn yield monosaccharides, SCFAs (i.e., butyrate (15%), acetate (60%), and propionate (25%)) and gases (i.e., methane and carbon dioxide) [43]. On the one hand, acetate and propionate are taken up by the liver through the portal vein where they are used as substrates for lipid, glucose, and cholesterol metabolism. Indeed, acetate is a precursor for cholesterol synthesis and lipogenesis while propionate is a gluconeogenic substrate [44]. On the other hand, butyrate plays a crucial role in maintaining tissue barrier function [45] and regulating gene expression and immunoregulation [46]. SCFAs are also involved in the colonic homeostasis, stimulating the proliferation and differentiation of epithelial cells, the absorption of salts and water, the maintenance of mucosal integrity, and decreasing inflammation [47,48]. Additionally, SCFAs can exert other beneficial effects, acting as histone deacetylase inhibitors, playing a crucial role in epigenetic regulation and acting as anti-cancer agents [16,49]; and increasing transit time and satiety by activating hormones such as glucagon-like peptide 1, peptide YY, and leptin via their endogenous receptors Free Fatty Acid Receptors 2 (FFAR2) and 3 (FFAR3) [50]. The types and amounts of SCFAs are mainly determined by the composition of intestinal microbiota and by how much carbohydrates are consumed [51]. Consequently, changes in the type and quantity of non-digestible carbohydrates in the human diet influence the bacterial populations detected in feces [52]. 

#### 3.1.3. Prebiotics

Prebiotics are non-digestible (by the host) food ingredients that have a beneficial effect through their selective metabolism in the intestinal tract [53]. Microbiota-accessible carbohydrates (MACs) are carbohydrates that are resistant to digestion by a host’s metabolism, and are made available for gut microbiota as prebiotics to metabolize into SCFAs [54]. Sonnenburg et al. [54] demonstrated that a low-MAC diet leads to an increase of *Bacteroides thetaiotaomicron*degrading intestinal mucus glycans. Indeed, these bacteria have the capacity to use host mucus glycans when dietary MACs are scarce, [24] leading to intestinal barrier thinning. Singh et al. [55] demonstrated that, in high-fat-fed male rats, inulin dose-dependently altered fecal microbiota by suppressing the number of Firmicutes (*Roseburia*, *Clostridium* clusters I, IV, and XIV) and promoting the number of *Bifidobacterium* spp. and Bacteroidetes. Vandeputte et al. [56] assessed the effects of inulin consumption on stool frequency in healthy adults with mild constipation, and detected specific inulin-induced changes in relative abundances of *Anaerostipes, Bilophila,* and *Bifidobacterium*. The observed decrease in *Bilophila* abundance following inulin consumption was associated with both softer stools and a favorable change in constipation-specific quality-of-life measures [56]. Therefore, all these dietary prebiotics interventions’ effects on the colon microbiota represent a promising novel target for mechanistic research.

### 3.2. Proteins and Gut Microbiota

The fermentation of amino acids occurs in the distal colon by major microbial phyla including Firmicutes, Bacteroidetes, and Proteobacteria. The proteolytic fermentation produces less SCFAs than saccharolytic fermentation, but also branched-chain fatty acids BCFAs (e.g., isobutyrate, 2-methyl butyrate, isovalerate) and potentially toxic substrates such as ammonia, the amines of which include nitrosamines and trimethylamine N-oxide [57]. Scott et al. demonstrated that aerobic genera such as *Escherichia*, *Pseudomonas*, *Proteus*, and *Klebsiella* were able to produce nitrosamine [57]. 

The effects of proteins on gut microbiota composition vary according to the protein type. The consumption of animal-based proteins—particularly from red meat and dairy products—may lead to an increase in abundance of bile-tolerant anaerobic bacteria such as *Bacteroides*, *Alistipes*, and *Bilophila* [58,59]. These gut microbiota alterations induce an increase of trimethylamine N-oxide (TMAO), a compound known for its proatherogenic potential [60], playing a role in cardiovascular diseases. Another study [61] showed that high consumption of animal-based proteins might increase the risk of inflammatory bowel diseases (IBDs) through an accrued production of hydrogen sulfide (H_2_S) by sulfate-reducing bacteria (SRB; e.g., *Desulfovibrio* spp.) from dietary inorganic sulfur and sulfated amino acids (i.e., methionine, cysteine, cysteine, and taurine). Moreover, animal-based protein fermentation decreased *Bifidobacterium* abundance and SCFA production, potentially increasing the risk of IBD [62]. 

On the other hand, one study [63] demonstrated that the consumption of vegetal proteins such as pea proteins increased gut-commensal *Bifidobacterium* and *Lactobacillus* and decreased pathogenic *Bacteroides fragilis* and *Clostridium perfringens.* Moreover, the supplementation of soy protein concentrates, after a Western-style diet for 3 weeks, led to significant increases in *Bifidobacteriaceae*, *Clostridiales*, and *Deferribacteraceae* abundances and decreases in Bacteroidetes levels in a Golden Syrian hamster model [64]. A beneficial impact of soy consumption on gut microbiota could be enhanced by soy isoflavones [65], while this benefit could be counterbalanced by a detrimental effect of soybean saponins on gut barrier [66]. Finally, the fermentation of plant-based protein may be associated with an increase of *Bifidobacterium* and *Lactobacillus* abundance, stimulating SCFA production [62].

### 3.3. Fats and Gut Microbiota

Dietary fat quantity and quality influence gut microbiota composition [67]. Dietary fatty acids can be divided into saturated (SFAs), monounsaturated (MUFAs) and polyunsaturated fatty acids (PUFAs) according to the presence of double bonds between carbon molecules.

Mammalian animal products are the main source of SFAs. Several animal studies [68,69] described a decrease in Bacteroidetes and an increase in Firmicutes and Proteobacteria in mice fed with a high-fat diet (HFD)—specifically SFAs. Interestingly, these changes may be gradually reverted by a control diet [68,69]. Another study [70] also showed a decrease of *Bacillus bifidus* in mice fed an HFD. Therefore, high intake of dietary fats and particularly SFA could lead to intestinal dysbiosis.

**Figure 1:**
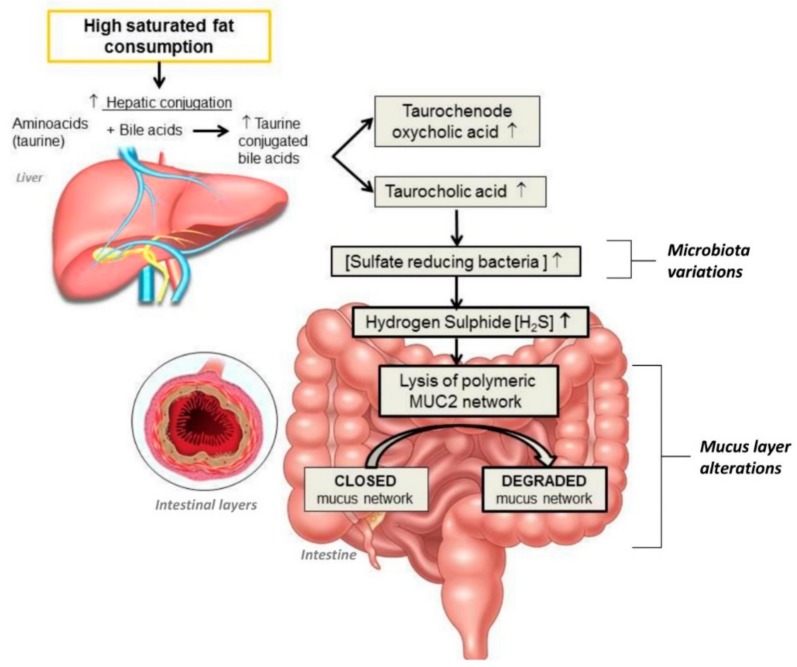
Impact of a high-fat diet on gut microbiota and mucus barrier. [ ], concentrations; ↑, increase; MUC2, Mucin 2.

Furthermore, HFD-induced dysbiosis could lead to intestinal barrier alterations. Indeed, sulfate-reducing bacteria (SRB) are more abundant in hosts consuming high-fat diets such as milk fat [71,72]. The high concentration of sulfide produced by particular SRB such as *Bilophila wadsworthia* may reduce disulfide bonds in the mucus, lysing the network of polymeric proteins MUC2 (oligomeric mucus gel-forming) secreted by goblet cells and having a key role in mucus layer stability and mucosal repair [73]. Recent lines of evidence have indicated that consumption of a high-SFA diet can stimulate the production of SRB, causing a defective mucus layer and increasing gut inflammation [73], colitis scores [73], and IBD [74,75]. The effects of HFD on gut microbiota are illustrated in Figure 1. 

MUFAs such as the oleic acid present in extra virgin olive oil (EVOO) are among the main components of the “Mediterranean diet”. Extra virgin olive oil consumption has been demonstrated to hold most of the cardioprotective properties of the Mediterranean diet, and it is particularly advisable to reduce the risk of coronary heart disease [76]. However, its antioxidant and anti-inflammatory properties do not seem to be as related to the MUFA oleic acid as to its phenolic compounds [77]. A recent systematic review [78] showed that high-MUFA diets have no effect on richness/diversity indexes, phylum distribution, or Bacteroidetes-to-Firmicutes ratio. At family and genus level, MUFA-rich diets could be positively correlated with *Parabacteroides*, *Prevotella,* and *Turicibacter* genera and *Enterobacteriaceae* family, and with a lower number of *Bifidobacterium* genus. Moreover, the abundance of *Blautia* detected in high body mass index (BMI) individuals could be positively associated with MUFA serum metabolites, whereas the abundance of the phylum *Tenericutes*, correlating with lower triglyceride levels, was negatively associated with MUFA metabolites [78]. 

PUFAs are largely found in sunflower, soybean, and corn oil, as well as nuts and seeds. They are subdivided into omega-3 PUFAs (including linolenic acid) and omega-6 PUFAs (including linoleic acid). PUFAs are also called “essential fatty acids” since they cannot be synthesized by the human body and need to be obtained from the diet. Omega-3 PUFAs, especially found in fatty fish, can exert a positive action by restoring a healthy microbiota composition and increasing the production of anti-inflammatory compounds. Several studies have demonstrated that omega-3 PUFAs are able to restore the ratio of Firmicutes/Bacteroidetes and increase *Lachnospiraceae* taxa [79,80,81]—both of which are associated with increased production of the anti-inflammatory SCFA butyrate [79,80,81]. 

Unfortunately, the increased dietary intake of omega-6 PUFAs and the decreased intake of omega-3 PUFAs have dramatically shifted the human evolutionary ratio of ~1:1 to the modern ratio ranging from 10:1 to 50:1 [82]. This effect has been correlated to the epidemic spread of cardiovascular and chronic diseases [83,84]. A high omega-6/omega-3 PUFA ratio, predominant in the Western diet, has been related to an enhanced gut barrier permeability and metabolic endotoxemia through a gut-microbiota-driven mechanism [82]. Restoring this ratio towards a major uptake of omega-3 PUFAs could ameliorate gut microbiota composition and consequently reduce metabolic endotoxemia. 

A distinct family of PUFAs are the conjugated linoleic acids (CLAs), of which the most naturally abundant are 18:2*cis*-9, *trans*-11 (9,11 CLA, or rumenic acid), and 18:2*trans*-10, *cis*-12 (10,12 CLA). These types of PUFA derive from the biohydrogenation of linoleic acid by ruminant bacteria expressing linoleic acid isomerase. For this reason, CLAs are found in ruminant animal food products such as beef, lamb, butter, and dairy products. Interestingly, several lines of evidence have shown anti-atherosclerotic, anti-obesogenic, and anti-cancer properties of CLAs, which are considered “Generally Regarded as Safe (GRAS)” by the Food and Drug Administration (FDA) [85]. It has been demonstrated in murine models that diet supplementation with 10,12 CLA can promote notable changes in gut microbiota composition at the phylum level such as a decrease of Firmicutes and an increase of Bacteroidetes [86] and at the species level with an enrichment of *Butyrivibrio*, *Roseburia*, and *Lactobacillus*, resulting in significant elevations of the SCFAs butyrate in the feces and acetate in plasma [87]. Such effects on gut microbiota composition could partially explain the beneficial properties attributed to CLAs.

### 3.4. Salt and Gut Microbiota

The World Health Organization recommends a maximum salt intake of 5 g/day [88]. A high-salt diet (HSD) is one of the major risk factors in the development of hypertension [89], kidney injury, and cardiovascular diseases (CVDs). HSD is also associated with an increased risk of gastric cancer by directly damaging gastric mucosa, leading to hyperplasia of the epithelium [90]. Moreover, considering *H. pylori* CagA-positive strains as markers of gastric cancer risk, Loth et al. showed that, when exposed to HSD, gastric *H. pylori* changed its virulence by inducing the expression of CagA [91].

Regarding gut microbiota, several studies in mice models demonstrated that HSD is associated with a decreased abundance of *Lactobacillus* spp. [92,93,94], *Oscillibacter* [91], *Pseudoflavonifractor* [91], *Clostridium* XIVa [91], *Johnsonella* [94], and *Rothia* [94], and an increased abundance of *Parasutterella* spp. [56,91], *Erwinia genus* [95], *Christensenellaceae* [95] and *Corynebacteriaceae* [95], *Lachnospiraceae* [93], and *Ruminococcus* [93]. In particular, *Lactobacillus* spp. reduction associated with HSD increased Th17 cells, pro-inflammatory hallmarks of many inflammatory diseases [92]. Miranda et al. [92] went further, showing that the exacerbation of colitis in mice induced by HSD was associated with a reduction in *Lactobacillus* spp., leading to the alteration of protective SCFA production, and hypothesizing that these changes alter gut immune homeostasis and lead to increased vulnerability to inflammatory insults [92]. HSD may result in alterations of gut microbiota composition, with a possible increase of Firmicutes/Bacteroidetes ratio, which may alter SCFAs production associated with modifications of gut permeability and immune homeostasis [92]. 

### 3.5. Food Additives and Gut Microbiota

With the development of the ultra-processed foods mainly characterizing the Western-type diet, the number of food additives such as non-nutritive sweeteners and emulsifiers approved for alimentary use by the industry has been soaring over the past few decades [96]. Artificial sweeteners are incorporated into almost all processed foods, often to aid stability and shelf life, and to improve taste and texture. Many reports [97,98] have shown that artificial sweetener consumption could alter gut microbiota and induce microbiota-mediated adverse effects in the host (e.g., glucose intolerance). Indeed, Suez et al. [98] demonstrated that non-caloric artificial sweeteners (NASs) altered microbial metabolic pathways and linked these changes to host susceptibility to metabolic disease. In this study, a proportion of healthy volunteers who did not normally consume NAS and who were given saccharin for one week at a dose of 5 mg/kg developed poorer glucose tolerance. The analysis of “NAS” responders’ stools showed an increase of *Bacteroides* spp. and *Lactobacillus* spp. and a decrease of *Clostridiales* spp. Several studies [97,99] confirmed that NAS intake increased the abundance of Bacteroides and some *Clostridiales* spp., and decreased the abundance of some *Clostridiales* spp., *Bifidobacterium*, and *Lactobacillus*. Another study by Palmnäs et al. reported significant gut microbiota changes in rats drinking water with low doses (5–7 mg/day) of aspartame, such as an increased abundance of *Enterobacteriaceae* and *Clostridium leptum* along with elevated fasting glucose levels and impaired insulin responses [100]. As these microbiota variations could cause glucose intolerance, these studies raise interesting questions on the harmlessness of artificial sweeteners and the functional meaning of gut microbiota. 

Unlike other low-calorie sweeteners, steviol glycosides (extracted from stevia leaf) have no reported consistent microbial changes on anaerobic fecal cultures taken from healthy human subjects [101]. Specifically, no changes among *Bacteroidaceae* and *Clostridia* species were reported [102]. To date, there is no evidence that steviol glycosides adversely impact colonic bacteria [103].

In addition to artificial sweeteners, dietary emulsifiers such as lecithins, mono- and diglycerides of fatty acids, could increase bacterial translocation across epithelia in vitro, promoting systemic inflammation, altering microbiota localization and composition [104]. Emulsifier intake decreased gut microbial diversity, decreasing *Bacteroides* abundance and increasing Verrumicrobia (*Akkermansia muciniphila*), Proteobacteria abundances, and mucolytic operational taxonomic units (OTUs) including *Ruminicoccus gnavus*. These microbiota alterations led to dysbiosis and chronic gut inflammation, promoting colitis and metabolic syndrome [104]. 

### 3.6. Micronutrients and Gut Microbiota

Micronutrients play an important role in shaping the gut microbiota, which in turn is an efficient player in mediating their protective health effects [105]. 

Vitamins and minerals play a key role in regulating energy metabolism, cellular growth and differentiation, and immune functions. Various vitamins, such as thiamine, riboflavin, niacin, biotin, pantothenic acid, and folate (B vitamins), as well as vitamin K, can be synthesized by the fecal microbiota [106]. Indeed, Magnúsdóttir [107] assessed that B-vitamins are biosynthesized by the cooperation of various gut bacteria. For example, all bacteria from the phyla Bacteroidetes, Fusobacteria, and Proteobacteria possessed the necessary pathways for riboflavin and biotin biosynthesis. Moreover, all the Fusobacteria were predicted to be producers of vitamin B12 while Bacteroidetes appeared to be the phylum with the greatest number of predicted B vitamin producers. On the other hand, several studies demonstrated that vitamins such as vitamin D could impact gut microbiota composition. For example, it has been demonstrated that vitamin D had a positive effect on IBD patients by modulating the gut microbiome and increasing the abundance of potentially beneficial bacterial strains [108]. Furthermore, in a recent study of infant gut microbiome at age 3–6 months, researchers showed that vitamin D was associated with increased *Lachnobacterium* but decreased *Lactococcus* [109], and suggested that these correlations could have possible long-term implications for immune system modulation and asthma/allergic disease incidence.

Other antioxidant vitamins such as carotenoids, responsible for the yellow, orange, and red colors of many fruits and vegetables, could influence gut microbiota composition. A recent phytotherapy study on humans [110] identified lutein as a component of blackcurrant extract powder and demonstrated that lutein significantly promoted the growth of bifidobacteria and lactobacilli and reduced other bacteria populations, such as *Bacteroides* spp. and *Clostridium* spp. On the other hand, serum carotenoid levels could be impacted by gut microbiota composition. Indeed, Karlsson et al. [111] conducted a study to identify the association between alterations of the gut metagenome with atherosclerosis and suggested that high levels of beta-carotene in the serum of healthy controls could be due to the potential production of this anti-oxidant by the gut microbiota [111]. This study suggested that the anti-inflammatory effects of beta-carotene are mediated by the gut microbiota.

Like vitamins, metals are involved in numerous bacterial physiological processes impacting the gut microbiota [16]. Indeed, in mice colonized with the well-known nosocomial pathogen *Clostridium difficile*, excess dietary zinc severely exacerbated *C. difficile*-associated disease by increasing toxin activity and altering the host immune response [112]. Iron is another key element involved as a cofactor in iron-containing proteins for redox reactions, metabolic pathways, and electron transport chain mechanisms [113]. Iron availability influences the composition of the microbiota. Indeed, Constante et al. [114] demonstrated in mice that a heme-rich diet decreased microbial diversity, increased the abundance of Proteobacteria, and reduced the abundance of Firmicutes. Furthermore, the heme-rich diet may stimulate the growth of bacteria-coding genes related to heme uptake and release from red blood cells [114].

### 3.7. Polyphenols and Gut Microbiota

More than 10,000 polyphenol compounds have been identified in various plants and foods, such as fruits, vegetables, tea, medical plants, microalgae, herbs, seeds, and cereals, and in beverages such as coffee, tea, cocoa, and wine [115]. Some edible and wild fruits such as grape, olive, blueberry, sweetsop, mango, and citrus fruits contain high contents of polyphenols [116]. As these compounds are known to be implicated in preventing CVD [117], diabetes, obesity, and many other diseases [118], they currently represent a topic of growing interest for the scientific community. However, the absorption and bioavailability of these compounds in humans remain unclear and controversial. Nevertheless, there is strong agreement among researchers that reciprocal interactions of gut microbiota and phenolic compounds have an important impact on the bioavailability of phenolic compounds [119].

Over the past decade, several studies [120,121,122,123] demonstrated that phenolic compounds can alter the gut microbiota, resulting in a greater abundance of beneficial microbes. For example, quercetin supplementation resulted in an altered composition of gut microbiota at different taxonomic levels, including the relative Firmicutes:Bacteroidetes ratio and inhibiting the growth of bacterial species associated with diet-induced obesity such as *Erysipelotrichaceae*, *Bacillus* spp., and *Eubacterium cylindroides* [120]. The anthocyanins significantly stimulate the growth of *Bifidobacterium* spp., *Lactobacillus* and *Enterococcus* spp. [121], suggesting that anthocyanins may positively select for beneficial members of the gut microbial community [121]. As phenolic compounds could exert prebiotic activity, it is crucial to understand their inhibitory or stimulatory effects on beneficial or pathogenic bacteria [119].

At the same time, gut microbiota can modulate the transformation of phenolic compounds into smaller metabolites, influencing their bioavailability and modifying their properties [119]. The gut microbiota plays a key role in modulating the bioavailability of pro-anthocyanidins [124]. Pro-anthocyanidins could exert local beneficial biological actions on colonic epithelial cells, resulting in protective effects against inflammation-mediated diseases including colorectal cancer [124].

Phenolic compounds could affect gut microbiota composition [120,121,122,123]. Moreover, the gut microbiota has an impact on the biotransformation of phenolic compounds [119]. Although reciprocal interactions have been demonstrated [119,120,121,122,123], they are not yet understood.

## 4. Effects of Dietary Habits on Gut Microbiota

**Figure 2:**
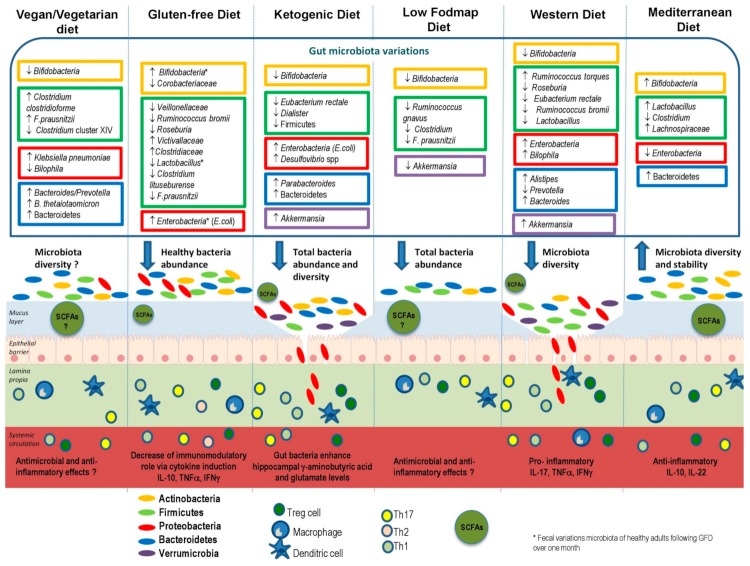
Effects of different types of diet on gut microbiota, mucus layer, and immune cells. Bacteria species variations are indicated in rectangular frames. The arrows pointing up or down respectively indicate an increase or decrease of bacteria abundance. Each color of the rectangular frames represents one phylum: yellow for Actinobacteria, green for Firmicutes, red for Proteobacteria, blue for Bacteroides, and purple for Verrumicrobia. In the illustration of the intestinal epithelium, oval shapes represent microbiota. Each color represents one phylum. Abbreviations: FODMAP: fermentable oligo-, di-, mono-saccharides, and polyols; GFD: gluten-free diet; SCFAs: short-chain fatty acids.

Since our dietary habits are the result of a specific mixture of micro and macronutrient amounts, continuously and indefinitely delivered to our gut ecosystem, we considered it useful to evaluate the impact of innovative and current dietary habits on gut microbiota associated with host mucosal barrier and immune functions. Figure 2 illustrates the effects of different types of diet on commensal bacterial species, mucus layer, and lamina propria hosting immune cells. 

### 4.1. Vegan and Vegetarian Diets

Unlike omnivores, vegetarians refrain from consuming all types of meat and seafood. Vegans represent a subgroup of vegetarians, excluding also animal products such as eggs, milk and dairy products, and honey from their diet. Several studies have compared the gut microbiota of omnivores, vegetarians, and vegan individuals. Some of them [125,126] showed higher ratios of *Bacteroides/Prevotella* [125], *Bacteroides thetaiotaomicron* [125], *Clostridium clostridioforme* [125], *Klebsiella pneumoniae* [126], and *Faecalibacterium prausnitzii* [125] and lower ratios of *Clostridium* cluster XIVa [125] and *Bilophila wadsworthia* [126] in vegetarians and vegans compared to omnivores. Another study [127] demonstrated both vegans and vegetarians had lower counts of *Bifidobacterium* and *Bacteroides* species, but the quantification of fecal SCFA levels and methane production by breath revealed no difference between vegans and omnivores, demonstrating that vegan and vegetarian diets could decrease gut microbiota diversity but not decrease SCFAs and methane levels. A recent cross-sectional study [128] suggested that vegan and vegetarian diets influence the microbiota but do not allow conclusions to be drawn about gut microbial composition. Indeed, all these results should be interpreted cautiously due to different methodologies for microbiota identification, varied sample sizes, and the influences of geographical origin, age, gender, and body mass [129]. Additionally, the effects of polyphenols, which are abundant in plant foods and thus in vegan and vegetarian diets, on gut microbiota modulation should be considered. Indeed, these components increase the abundance of beneficial bacteria such as *Bifidobacterium* and *Lactobacillus*. However, further studies are warranted to clarify the complex mechanisms and interrelationships between vegan/vegetarian diets and gut microbiota.

### 4.2. Gluten-Free Diet (GFD)

Celiac disease (CD) is an autoimmune-mediated disease in which gluten sets off intestinal inflammation. In the 1960s, the gluten-free diet (GFD) was recognized as a potential cure, restoring the normal intestinal mucosa in coeliac patients [130]. At present, GFD is still known as the unique therapy in CD and the most commonly adopted special diet worldwide [131].

De Palma et al. [132] studied the gut microbiota of healthy subjects following a GFD over 1 month, and demonstrated a decrease of *Bifidobacterium*, *Clostridium lituseburense*, and *Faecalibacterium prausnitzii* proportions and an increase of *Enterobacteriaceae* and *Escherichia coli* counts after the GFD. Moreover, Bonder et al. [133] showed that the strongest gut microbiota variations in GFD occur in the family *Veillonellaceae*, whereby the abundance in the gut drops significantly in GFD. They also demonstrated a decrease of *Ruminicoccus bromii* and *Roseburia feces* in GFD while families *Victivallaceae*, *Clostridiaceae,* and *Coriobacteriaceae* increased in abundance in GFD. Sanz et al. [134] demonstrated that the fecal microbiota of healthy adults following a GFD over one month was eroded in healthy bacteria such as *Bifidobacterium*, *B. longum*, and *Lactobacillus*, and unhealthy bacteria increased (e.g., *Enterobacteriaceae*, in particular, *E. coli)*. 

Although most of these studies have important limitations including small sample sizes and the use of low-throughput techniques (e.g., culture techniques and non-sequencing-based molecular techniques [135]), a decrease of healthy bacteria such as *Bifidobacterium* and *Lactobacillus* has been demonstrated, leading to a diminution of SCFAs production and their beneficial metabolic and host immunity effects. Long-term GFD could constitute an environmental variable to be considered in treated CD patients for its possible effects on gut health, improving coeliac disease symptoms [132]. However, the increase of detrimental species such as *Enterococcus*, *Staphylococcus, Salmonella*, *Shigella*, and *Klebsiella* demonstrated by several studies [132,133,134,136] could influence the microbial profiles and impact the long-term homeostasis of the intestinal mucosa of healthy subjects [137]. 

### 4.3. Ketogenic Diet

The ketogenic diet (KD) is a high-fat, very-low-carbohydrate normocaloric diet used for drug-resistant epilepsy and GLUT1 Deficiency Syndrome [138]. In obese patients, KD seems to act as an efficient diet therapy for weight reduction [139]. However, maintaining body weight after weight loss is usually a major problem. Moreover, regarding the long-term impact of KD on cardiovascular risk factors, study results are controversial [140,141,142,143]. Some studies [141,143,144] demonstrated adverse events such as the development of non-alcoholic fatty liver disease (NAFLD) or insulin resistance.

The study of Tagliabue et al. [138] on patients treated with KD comparing their fecal microbiota composition before and after three months on the diet demonstrated that fecal microbiota increased in *Desulfovibrio* spp. involved in the exacerbation of gut inflammation. Another study [145] analyzed taxonomic changes in the children’s microbiota before and 3 months after starting KD, and demonstrated that alpha diversity did not change significantly during the diet. However, differences in both taxonomic and functional composition were detected, with a decrease in the abundance of bifidobacteria, *E. rectale*, and *Dialister* and an increase of *E. coli* during the intervention. Lindefeldt et al. [145] also showed, in children with refractory epilepsy following a week of KD, a reduction in the richness of gut microbiota with an increase of Bacteroidetes and reduction of Proteobacteria after KD. At the genus level, *Bacteroides*, *Bifidobacterium*, and *Prevotella* were increased after KD, while *Cronobacter* diminished [146]. Olson et al. [147] went further, showing an anti-seizure effect of KD in mice, mediated by *Akkermansia* and *Parabacteroides* involving changes in systemic gammaglutamylated amino acids and elevated hippocampal GABA/glutamate levels. 

KD results in a reduction of carbohydrate intake, and leads to a decrease of polysaccharide content associated with a decrease in beneficial gut microbiota bacteria such as *Bifidobacteria*. Moreover, all these studies showed microbial increases such as *Akkermansia* or *E. coli* during KD. This confirms a potential detrimental effect of an HFD on the gut mucus barrier, as described above. Therefore, although KD has a positive impact on a wide range of diseases [145], we raise a concern about the long-term effects of KD on the gut microbiota composition and consequently mucus layer homeostasis and immunity functions—more specifically in healthy subjects adopting KD for weight loss. Further studies are warranted to understand the role of gut microbiota variations during KD and its therapeutic effects.

### 4.4. High-Glucose or -Fructose Diets

The excess of sugar in modern dietary habits has been linked to obesity and several metabolic diseases, including diabetes mellitus type II, NAFLD, and cardiovascular diseases [148]. The impact of high-sugar diet on the gut microbiota has recently been elucidated by Do et al. [149]: the authors assigned six-week-old C57BL/6J mice to receive distinct dietary regimes such as normal diet (ND), high-glucose diet (HGD), and high-fructose diet (HFrD). After 12 weeks, HGD- and HFrD-fed mice showed lower microbial diversity (i.e., fewer operational taxonomic units and lower Shannon indices) than ND-fed mice, with a lower abundance of Bacteroidetes and an increased abundance of Proteobacteria at the phylum level, as well as an increase of the *Desulfovibrio vulgaris* species. These metabolic and microbial differences were accompanied by a significant (2.5-fold greater) increase in gut permeability. Consequently, the expression of inflammatory cytokines (TNF-alpha and IL-1beta) in the colon was higher in HGD- and HFrD-fed mice than in ND-fed mice. This evidence suggests that an HGD and/or HFrD can shape the gut microbiota, increasing the Firmicutes-to-Bacteroidetes ratio and the proportion of Proteobacteria—one of the best sources of LPS [150]. Moreover, these dietary regimens significantly alter gut permeability, boosting metabolic endotoxemia and systemic inflammation through modulation of the gut microbiota [105].

### 4.5. Low-FODMAP Diet

In 2004, the Monash University created the term “FODMAP” (fermentable oligo-, di-, mono-saccharides, and polyols) to describe a group of highly fermentable but poorly absorbed carbohydrates and polyols [151]. In recent years, this alternative diet gained visibility with physicians especially for its use as a treatment option in IBD and IBS [152,153]. Halmos et al. [154] demonstrated in IBS patients on a low-FODMAP diet, similar SCFA concentrations with a reduction of total bacterial abundance to 47% compared with a habitual diet. Several studies showed a decrease of *Clostridium* cluster IV [154], *Propionibacteriaceae* [155], mucus-associated *Akkermansia muciniphila* [154], *Ruminococcus gnavus* [115], and *Bifidobacteria* [154,156,157] in low-FODMAP diet compared to control diet. Indeed, a low-FODMAP diet could lead to a reduction of potential prebiotics (FOSs and GOSs), thus leading to a reduction in beneficial bacteria and fermentative effects. The integration of a low-FODMAP diet with probiotics seems to counteract gut microbiota imbalances and, in particular, restore *Bifidobacterium* levels [131]. Probiotics are live microorganisms that, when administered in adequate amounts, confer a health benefit on the host [158]. Each probiotic may have different characteristics, including variable effects on cytokines, host microbiota, and other potential targets [159]. Larger studies are needed to further understand the potential contributions of probiotics supplementation and long-term effects of low-FODMAP diets on intestinal microbiota composition. 

### 4.6. Western Diet

The Western diet (WD) is a dietary habit, as part of the Western lifestyle chosen by many people in developed countries, and increasingly in developing countries, associated with economic growth. The WD is enriched in total fat, animal proteins, and refined sugars. Martinez Medina et al. [160] demonstrated that a combination of high-fat/high-sugar diet led to dysbiosis with increased *Bacteroides* spp. and *Ruminococcus torques* in mice. A study [161] of European children fed with the WD and Burkina Faso (BF) children assuming a diet rich in millet/sorghum + local vegetables containing very few lipids and animal proteins revealed that Proteobacteria were more abundant in EU than in BF children. Moreover, the BF children’s microbiota was enriched with *Prevotella* and *Xylanibacter* compared to EU children’s microbiota. Another study [162] determined the microbiota composition of volunteers from Venezuela, Malawi, and the United States, and reported that irrespective of age, Malawian and Venezuelan microbiota compositions were similar, compared with the US American microbiota characterized by the least microbial diversity. These two studies demonstrated that the genus *Prevotella* was underrepresented in WD microbiota and could be a taxonomic discriminator. Furthermore, the WD based on animal protein consumption increases the abundance of bile-tolerant microorganisms such as *Alistipes, Bilophila*, and *Bacteroides* and decreases the levels of Firmicutes that metabolize dietary plant polysaccharides such as *Roseburia*, *Eubacterium rectale*, and *RRuminococcus bromii* [58]. Moreover, several studies suggest that the detrimental effects of WD on gut microbiota may also be driven by food additives, inducing dysbiosis and consequently adverse intestinal mucosal effects and inflammation [104]. Moreover, a large body of research supports the hypothesis that the WD, by causing dysbiosis in gut microbiota composition, is associated with obesity and metabolic diseases [163].

### 4.7. Mediterranean Diet

The concept of the Mediterranean diet (MD) was developed to reflect the typical dietary habits followed during the early 1960s by inhabitants of the Mediterranean basin, mainly in Crete, much of the rest of Greece, and Southern Italy [164]. The MD, centered around fruits, vegetables, olive oil, nuts, legumes, and whole grains, has been linked to a large number of health benefits, including reduced mortality risk and the prevention of many diseases such as CVD [165], diabetes [166], metabolic syndrome [167], cognitive impairment [168,169], and depression [169]. The MD is based on the regular consumption of MUFAs and PUFAs, polyphenols and other antioxidants, a high intake of prebiotic fiber and low-glycemic carbohydrates, and greater consumption of plant proteins than animal proteins. The recent study of Garcia-Mantrana et al. [170] indicated that a higher ratio of Firmicutes–Bacteroidetes was related to lower adherence to the MD, whereas the greater presence of Bacteroidetes was associated with lower animal protein intake. Furthermore, higher bifidobacterial counts and higher total SCFAs were related to greater consumption of plant-based nutrients, such as vegetable proteins and polysaccharides. Finally, they demonstrated that better adherence to the MD was associated with significantly higher levels of total SCFAs. De Filippis and colleagues [171] also reported that high-level consumption of plant-based foods and high-level adherence to an MD beneficially impact the gut microbiota and associated metabolomic profile [171]. Mitsou et al [172] confirmed these findings, with positive correlations between adherence to MD and increase of total bacteria, *Bifidobacteria*/*E. coli* ratio, the relative share of *Bacteroides*, *C. albicans*, and total SCFAs, as well as decrease of *E. coli* levels. All these findings demonstrated a link between adherence to the MD and improvements to the diversity and richness of gut microbiota. 

## 5. Conclusions

Food components have a key impact on the gut microbiota, influencing its composition in terms of richness and diversity. On the one hand, high intake of animal proteins, saturated fat, sugar, and salt could stimulate the growth of pathogenic bacteria to the detriment of beneficial bacteria, leading to potential alterations of the intestinal barrier. On the other hand, the consumption of complex polysaccharides and plant protein could be associated with an increase of beneficial bacteria quantity, stimulating SCFA production. Moreover, omega-3, polyphenols, and micronutrients appear to have the potential to confer health benefits via modulation of the gut microbiota. 

Dietary habits can strongly influence gut microbiota composition. The Westernization of the diet, including additives, may reduce gut microbial diversity in terms of phyla and genus leading to dysbiosis, alteration of barrier function and permeability, and abnormal activation of immune cells, leading to high incidences of chronic diseases. Although elimination diets such as low-FODMAP and GFD can improve the symptoms of some diseases like IBS and CD in selected patients, the long-term effects on gut microbiota require elucidations. To date, the Mediterranean diet remains the evergreen solution to optimally modulating microbiota diversity and stability as well as the regular permeability and activity of immune functions of the human host. Modifying dietary habits and adopting a MD may be the solution to prevent microbiota dysbiosis, and consequently, to prevent many GI and neurological disorders [13]. 

Future advances on the knowledge of the interactions between food compounds and specific intestinal bacteria would lead to a better understanding of both positive and negative interactions with dietary habits. A novel nutritional approach may be adopted by building a personalized diet subsequent to microbiota analyses, in order to modulate and restore a healthy gut microbiota.
